# Phytase overexpression in Arabidopsis improves plant growth under osmotic stress and in combination with phosphate deficiency

**DOI:** 10.1038/s41598-018-19493-w

**Published:** 2018-01-18

**Authors:** Nibras Belgaroui, Benoit Lacombe, Hatem Rouached, Moez Hanin

**Affiliations:** 1 Laboratoire de Biotechnologie et Amélioration des Plantes, Centre de Biotechnologie de Sfax, BP “1177”, 3018 Sfax, Tunisia; 2 BPMP, CNRS, INRA, Montpellier SupAgro, Univ Montpellier, Montpellier, France; 3 Unité de Génomique Fonctionnelle et Physiologie des Plantes, Institut Supérieur de Biotechnologie, Université de Sfax, BP “1175”, 3038 Sfax, Tunisia

## Abstract

Engineering osmotolerant plants is a challenge for modern agriculture. An interaction between osmotic stress response and phosphate homeostasis has been reported in plants, but the identity of molecules involved in this interaction remains unknown. In this study we assessed the role of phytic acid (PA) in response to osmotic stress and/or phosphate deficiency in *Arabidopsis thaliana*. For this purpose, we used *Arabidopsis* lines (L7 and L9) expressing a bacterial beta-propeller phytase PHY-US417, and a mutant in *inositol polyphosphate kinase 1* gene (*ipk1-1*), which were characterized by low PA content, 40% (L7 and L9) and 83% (*ipk1-1*) of the wild-type (WT) plants level. We show that the *PHY*-overexpressor lines have higher osmotolerance and lower sensitivity to abscisic acid than *ipk1-1* and WT. Furthermore, *PHY*-overexpressors showed an increase by more than 50% in foliar ascorbic acid levels and antioxidant enzyme activities compared to *ipk1-1* and WT plants. Finally, *PHY*-overexpressors are more tolerant to combined mannitol stresses and phosphate deficiency than WT plants. Overall, our results demonstrate that the modulation of PA improves plant growth under osmotic stress, likely via stimulation of enzymatic and non-enzymatic antioxidant systems, and that beside its regulatory role in phosphate homeostasis, PA may be also involved in fine tuning osmotic stress response in plants.

## Introduction

Soil salinity adversely affects plant growth and development and constitutes *de facto*, one of the major environmental constraints limiting agricultural production worldwide^1^. The ionic component of salt stress could be attributed to the direct cytotoxic effects of sodium (Na^+^) that impairs numerous metabolic processes mainly by competing with an essential cation potassium (K^+^), which is required for several biochemical reactions and protein synthesis^1,2^. This stress affects profoundly an essential physiological process in plant, photosynthesis^1,3,4^. Salinity stress also induces the accumulation of reactive oxygen species (ROS), known to be detrimental to cells since they cause oxidative damage to membrane lipids, proteins, and nucleic acids^5,6^. To scavenge high ROS levels, plant cell activates enzymatic (i.e. superoxide dismutase (SOD), peroxidase (POX), catalase (CAT)) and non-enzymatic (i.e. phenolics, flavonoids, tocopherols, ascorbate (AsA) and glutathion (GSH)) antioxidant systems^5,7,8^. The accumulation of compatible solutes such as proline and glycine betaine^9,10^ has been also proposed to be part of cellular osmoprotection mechanisms developed by plants to cope with the salinity stress^11,12^. Recent progress towards understanding salt and osmotic stress tolerance mechanisms, revealed the presence of complex interconnecting molecular signalling pathways, between salt and osmotic stress responses and nutrients homeostasis, such as interaction between salt stress response and phosphate (Pi) homeostasis, to name only few^13–15^. Therefore, engineering osmotolerant crops should take into account the existence of such interaction and the identity of key genes and metabolites that mediate this osmotic stress tolerance.

M*yo*-inositol-1,2,3,4,5,6-hexa*kis*phosphate (InsP_6_) known also as phytic acid (PA) is a ubiquitous key component of eukaryotic cells where it regulates many cellular functions^16^. In plants, PA is the main phosphorus (P) storage form in plant seeds (for review, see^17^). During germination, PA is degraded through the action of phytases to remobilize Pi, likely to support seedling growth^18^. Two major PA biosynthesis pathways have been reported in higher plants, namely the lipid-dependent and the lipid-independent signaling pathways (for review see^19,20^). These two pathways differ essentially in their early intermediate steps leading from *myo*-inositol (Ins) to *myo*-inositol trisphosphates InsP_3_^19^. Myo-inositol synthesis begins with conversion of D-glucose-6-phosphate to *myo*-inositol-3-phosphate (Ins(3)P_1_) by the action of D- *myo*-inositol-3-phosphate synthase (MIPS). Ins(3)P_1_ is then dephosphorylated to yield *myo*-inositol under the hydrolytic activity of inositol monophosphate phosphatase (IMP).

In the lipid-dependent pathway, Ins is first transformed into phosphatitdylinositol (PtdIns), from which the Ins headgroup of PtdIns is phosphorylated to give rise to PtdIns(4, 5)P2. Then, PtdIns(4, 5)P2 is hydrolyzed via the action of phospholipase C (PLC) to yield Ins(1, 4, 5)P3 that can be stepwise phosphorylated into InsP_6_ by two inositol kinases, IPK2 an inositol polyphosphate multikinase 6-/3- kinase and IPK1 an inositol polyphosphate 2-kinase.

In the lipid-independent pathway, a sequential phosphorylation of the Ins ring to InsP_6_ occurs through the action of specific inositol phosphate kinases. The first step, consisting to the conversion of Ins to Ins(3)P_1_, is catalysed by *myo*-inositol kinase (MIK) (enzyme that reverse the action of IMP). The production of InsP_2_ from Ins(3)P_1_ requires a monophosphate kinase. Further phosphorylation steps to Ins trisphosphates such as Ins (3, 4, 6) P3, InsP4, InsP5 and on to phytic acid occur probably through the action of an inositol 1,3,4-trisphosphate 5-/6-kinase (ITPK) and the same suite of Ins polyphosphate kinases mentioned above^19,20^. While the first pathway operates in all plant tissues to generate “signalling InsP_6_” (and derivatives) regulating cellular processes^16,21–26^, the second predominates in storage tissues (seed, pollen, tubers) to form Pi reserves^19^.

In addition to its important cellular roles, PA is considered as anti-nutritional factor, preventing the uptake of essential minerals such as iron, zinc, magnesium and calcium since it acts as a strong chelator of not only cations^27,28^ but also proteins^29^. To overcome these problems, the reduction of seed phytate content can serve as a sustainable solution^19^ and several low phytic acid (*lpa*) mutants have been generated by classical mutations, or RNAi approaches (for review^17^). When mutations affect the first step of the of the biosynthetic pathway such is the case of down-regulation of MIPS encoding genes in rice^30,31^ or soybean^32–34^, the resulting *lpa* mutants are characterized by a decrease in InsP_6_ levels accompanied by a molar equivalent increase in inorganic Pi^19^. However, mutation that perturbs the end of the PA pathway, in one of the genes coding for inositol kinases (e.g. IPK1) causes a significant reduction in PA as observed in *Arabidopsis Atipk1.1*^35^, rice *Osipk1*^36^ and maize *Zm ipk1*^37,38^.

It has been reported that a number of *lpa* mutants are associated to negative phenotypic traits such as decreased seed germination, developmental delay, deficiency in grain filling, and stress sensitivity^13,39–48^. In *Arabidopsis*, while some *lpa* mutants (e.g. *ipk1*) showed higher degree of sensitivity to various abiotic stresses^13^, positive effects were observed on others, as it was the case for the *Arabidopsis* mutant (e.g. *Atmrp5-1*) that exhibited higher drought tolerance and less abscisic acid (ABA) sensitivity^42^. Alternative approaches to generate *lpa* mutants were successfully established by engineering plants to produce heterologous phytases (for review^17^). In most cases, the reduction of PA levels following its hydrolysis by expression of intracellular phytase genes is associated to an increase in free inorganic phosphate (Pi) and also several inositol phosphate derivates in plant tissues^49–51^. For instance, the over-expression of the bacterial phytase PHY-US417 in *Arabidopsis* led to 40% decrease in PA content and increase in foliar Pi concentration, associated to an improved plant growth capacity under P-limited conditions^51^.

In this study, we assessed the effect of a change in the cellular PA concentration on the salt and osmotic stress response of *Arabidopsis* plants. To do this, we used the PHY-US417 over-expressing lines (40% PA reduction), *ipk1* knock-out mutant (>80% PA reduction) and wild-type plants (Col-0). Our results demonstrate that a defined range of PA concentration decrease enhances significantly plant osmotic stress tolerance. With a 40% PA decrease, PHY-US417 over-expression promotes *Arabidopsis* growth under osmotic stress and this osmotolerance, is associated to higher antioxidant activities in the transgenic lines. Higher decrease of PA concentration (80%) such as observed in *ipk1-1* mutant is rather detrimental. In combination with our previous results^51^ showing that the overexpression of PHY-US417 improves the growth of plants under Pi deficiency, we demonstrate here that the PHY-US417 over-expression not only increases antioxidant activities and osmotic stress tolerance (individual stress), but also improves the plant capacity to tolerate combined osmotic stress and Pi deficiency. This constitutes a new and desired trait for biotechnological application in agriculture.

## Results

### Over-expression of PHY-US417 gene results in higher osmotic stress tolerance and lower abscisic acid sensitivity

**Figure 1:**
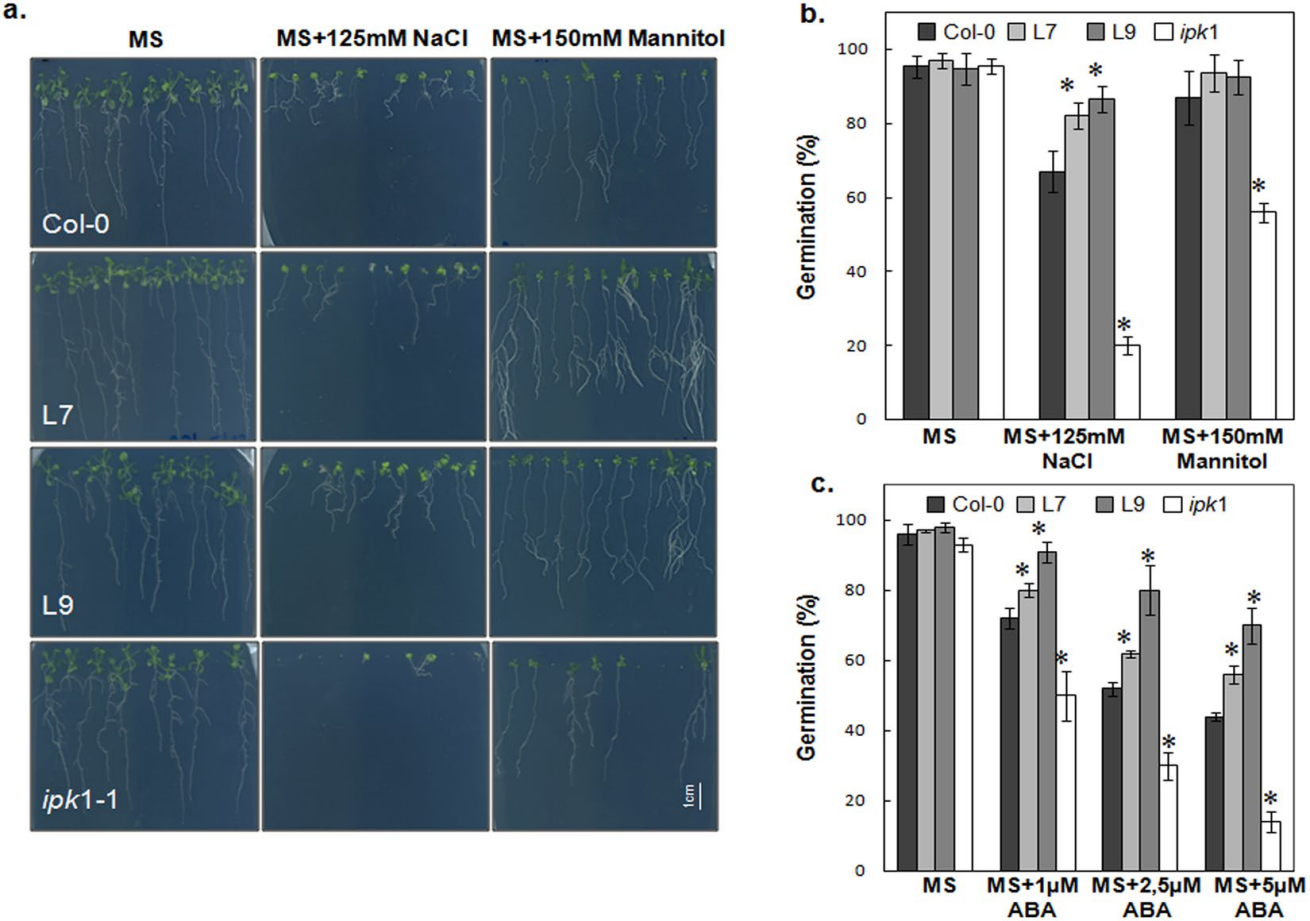
Response of wild-type (Col-0), PHY-expressing and *ipk1* KO *Arabidopsis* plants to salinity or osmotic stresses. (**a**) Effect of salt (125 mM NaCl) and mannitol (150 mM) treatments on root growth. Representative pictures were taken after 2 weeks of stress application. (**b**) Seed germination percentage on 125 mM NaCl and 150 mM mannitol supplemented MS media. (**c**) Seed germination percentage on MS supplemented with 1, 2.5 or 5 µM ABA. Vernalized seeds of WT, transgenic and mutant plants were sown in triplicates then monitored for 5 days. Seedlings with green cotyledons were counted for 100 seeds of each genotype. Data are mean ± SD of three independent experiments. Asterisks indicate a statistically significant difference (p < 0.05) relative to WT control (Tukey’s test).

Crosstalk between Pi homeostasis and response to salt and osmotic stresses was evoked in plants^13–15^. We set out to assess the involvement of phytic acid (PA) a key Pi-containing component, PA, in this connection. Therefore, we evaluated the responses of different *Arabidopsis* genotypes characterized by their differential capacity to accumulates PA subjected to salt (100–150 mM NaCl), and osmotic (150–300 mM mannitol), treatments. These plant genotypes included wild-type (WT, used as reference at 100% of PA), the phytase PHY-US417 over-expressing lines (L7 and L9) with 40% decrease and the *Arabidopsis ipk1-1* mutant exhibiting 83% decrease of PA^35,51^. Under standard growth condition (MS medium), no significant difference in either seed germination or root growth was observed between all tested genotypes. Under stress conditions, *ipk*1-1 mutant was more sensitive than WT to all treatments especially salt stress (MS + 125 mM NaCl) to which ~80% of seeds fail to germinate (Fig. 1b) and seedlings showing very short roots (Fig. 1a). By contrast to *ipk1-1*, this salt hypersensitivity was not observed in PHY-overexpressors (L7 and L9), which rather exhibit higher seed germination rates under salt stress compared to WT (82–86% for L7 and L9 versus 67% for WT). Most interestingly, L7 and L9 lines appear more tolerant to osmotic stress (Mannitol 150 mM) with significantly enhanced root growth, compared to WT (Fig. 1a). This result prompts us to check the sensitivity of the PHY-transgenic lines to ABA, which plays a central role in plant response to various stresses^52^. The seed germination rates of lines L7 and L9 in the presence of increasing ABA concentrations (1–5 μM) were compared to those registered in WT and *ipk1-1* mutant. Our results showed that in the presence of 1 μM of ABA, seed germination rates decreased up to 50% and 72% in *ipk1-1* and WT respectively (Fig. 1c), whereas L7 and L9 exhibit higher germination rates (up to 91%). On growth medium (MS) supplemented with higher concentrations of ABA, the seed germination continue to decrease to ~15% and ~45% in *ipk1-1* and WT in the presence of 5 μM ABA, respectively (Fig. 1c). However, under the same conditions, the germination rates were slightly reduced in L7 and L9 (58% and 70%). Therefore, the PHY-overexpressors appear less sensitive to ABA than WT.

**Figure 2:**
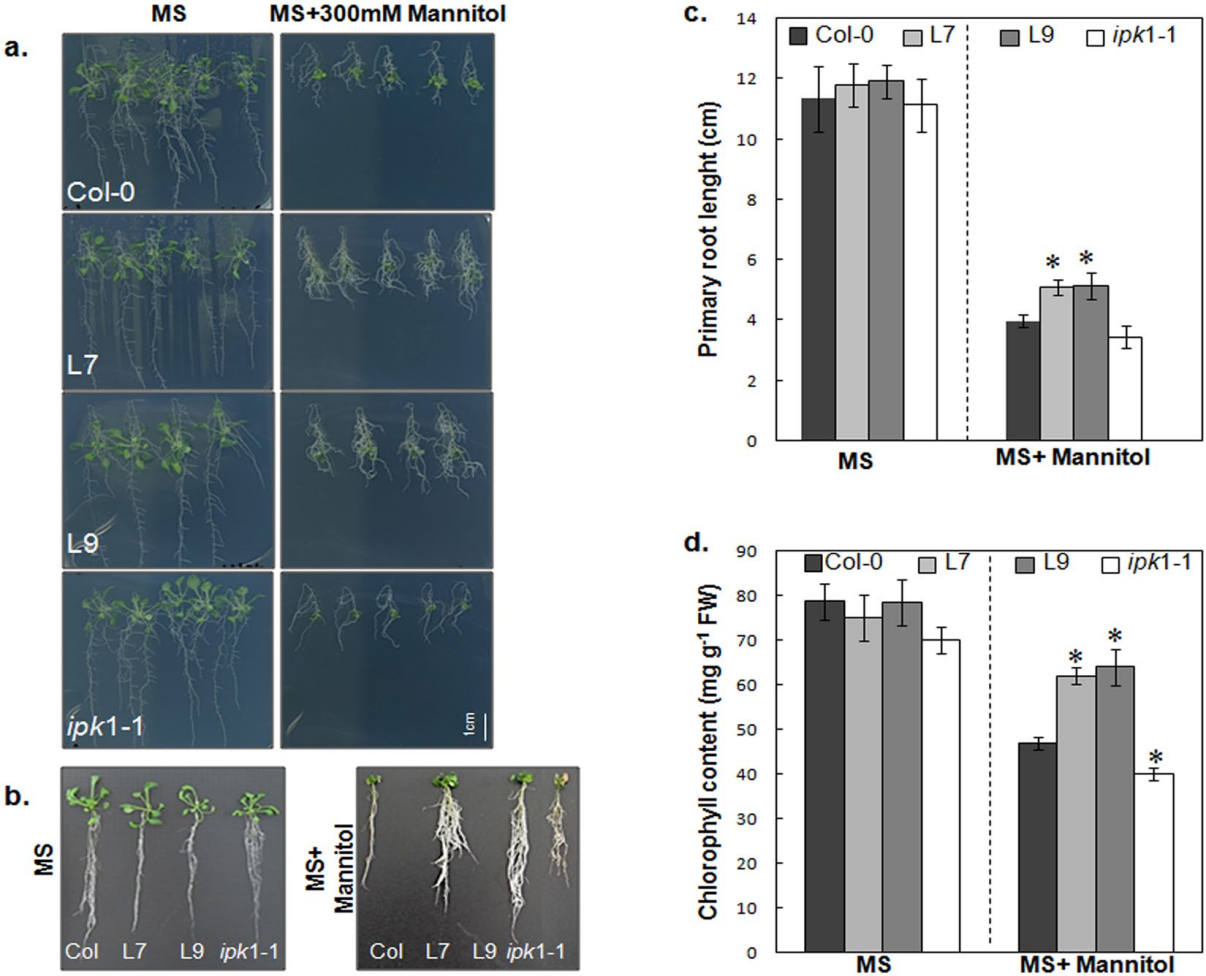
Effect of abiotic stresses on growth of wild-type (Col-0), PHY-transgenic lines and *ipk1-1* mutant. (**a**) one-week-old seedling germinated on MS medium were transferred to standard MS medium or MS medium supplemented with 300 mM mannitol for additional 2 weeks. Experiments were repeated at least three times. (**b**) Photographs of representative WT, transgenic lines (L7 and L9) and *ipk*1-1 mutant taken 2 weeks after transfer to MS medium or MS supplemented with 300 mM mannitol. (**c**) Primary root length of WT, transgenic lines (L7 and L9) and *ipk1-1* mutant grown as indicated in a. (**d**) Chlorophyll level in all tested plants under stress (300 mM mannitol) or unstressed conditions. Data are mean ± SD of three independent experiments. Asterisks indicate a statistically significant difference (p < 0.05) relative to WT (Col-0).

To better characterize L7 and L9 transgenic lines, *ipk1-1* mutant and wild-type plants, we assessed their root growth and chlorophyll contents when submitted to additional stress assays. Since the PHY-transgenic lines exhibit higher tolerance towards osmotic stress, we chose to perform our next assays on plants exposed only to mannitol treatments. One week-old seedlings grown on standard conditions (MS) were transferred to MS (control) or MS medium containing mannitol (300 mM). Under osmotic stress, the transgenic lines (L7 and L9) showed well developed root system with more lateral roots and longer primary roots (Fig. 2b,c), whose length exceed those of WT plants by ~28%. The *ipk1-1* mutant showed shorter roots in comparison to WT, under stress conditions (Fig. 2a and c).

Moreover, we registered higher chlorophyll contents in L7 and L9 lines (30–40% higher compared to WT and *ipk1-1* (Fig. 2d)) on (MS+300 mM Mannitol), suggesting that the transgenic lines may have retained higher photosynthetic capacity under osmotic stress.

Taken together, our results indicate that reduction of the PA levels through the phytase overexpression in *Arabidopsis* results in an enhanced osmotic stress tolerance and less sensitivity to ABA. Remarkably, a further decrease of PA as caused by *ipk1* disruption does not confer the same phenotype but rather leads to an exacerbated stress sensitivity, suggesting the existence of a critical threshold of PA to obtain salt and osmotic stress tolerance.

### The PHY-US417 overexpressing lines exhibit high level of ascorbic acid

**Figure 3:**
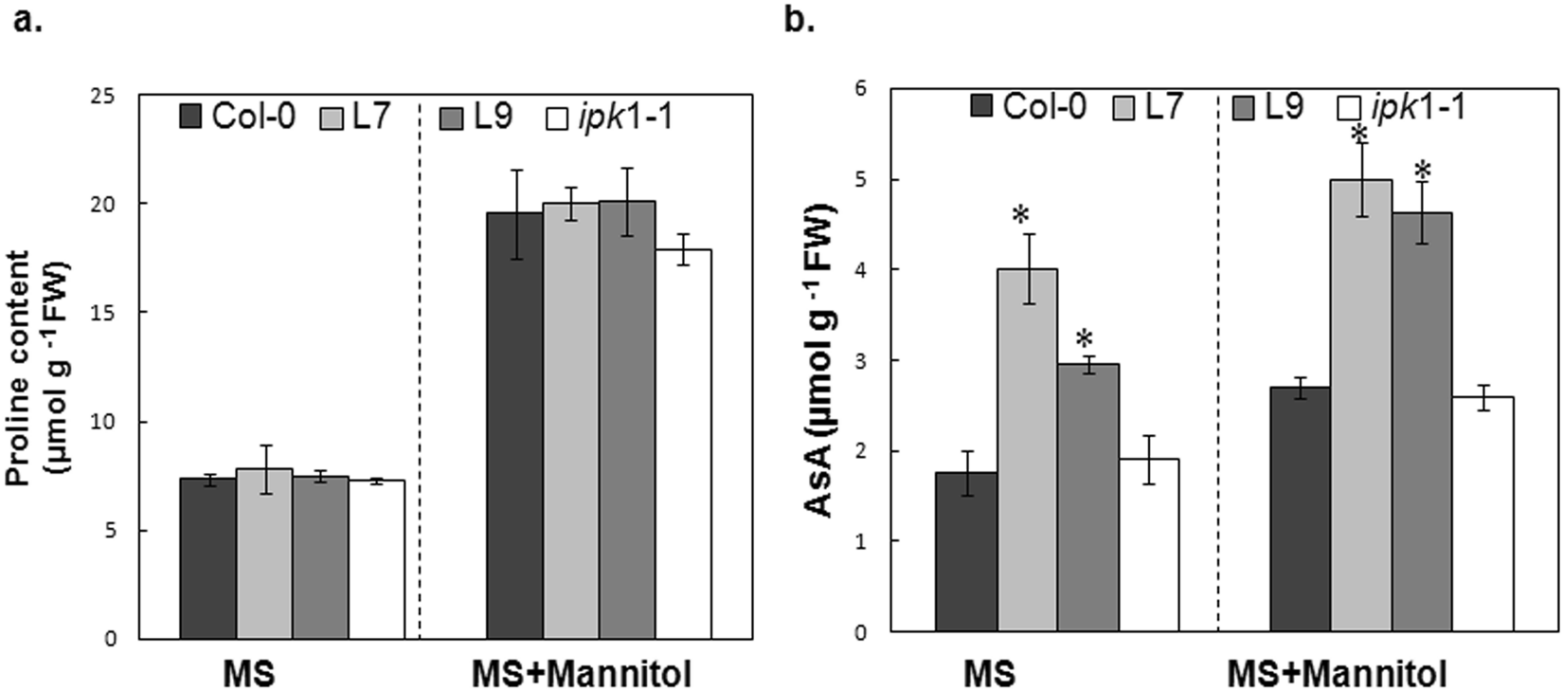
Proline and ascorbate levels in wild-type plants (Col-0), transgenic lines (L7 and L9), *ipk*1-1 mutant under osmotic stress. Seedlings were germinated on MS medium and then transferred to standard MS medium or MS medium containing 300 mM mannitol for additional 7d before proline (**a**) and ascorbate (AsA) (**b**) measurements. Mean ± SD is shown for three independent replicates. Asterisks indicate a statistically significant difference (p < 0.05) relative to WT (Col-0).

In literature, several osmolytes and antioxidants including proline and ascorbic acid (AsA), have been reported to be associated to plant stress responses^53,54^. Proline is an osmolyte that plays a crucial role in osmotic adjustment when plants are exposed to various stresses (drought, salinity, cold)^55,56^. AsA is a well-known antioxidant which protects plants against several abiotic stresses by scavenging free radicals and reactive oxygen species (ROS)^57–59^. Therefore, we monitored the accumulation of these two metabolites in 2-weeks-old WT, *ipk1-1* and the two PHY-overexpressing plants submitted to 300 mM mannitol. Our results didn’t reveal a significant difference in proline contents between all tested genotypes grown under either standard growth (MS) or stress conditions (Fig. 3a). However, interestingly, compared to WT plants, the PHY-transgenic seedlings appeared to accumulate higher level of AsA in the absence (1, 7 to 2, 3 fold increase) or in the presence of mannitol (1, 8 to 1, 6 fold increase) (Fig. 3b). No significant change of AsA was recorded in the *ipk1-1* mutant (Fig. 3b). This result strongly suggests that the constitutive expression of phytase PHY-US417 may participate in AsA synthesis/accumulation, which in turn can contribute into increasing osmotolerance of L7 and L9.

### The PHY-US417 transgenic lines exhibit enhanced ROS scavenging capability

**Figure 4:**
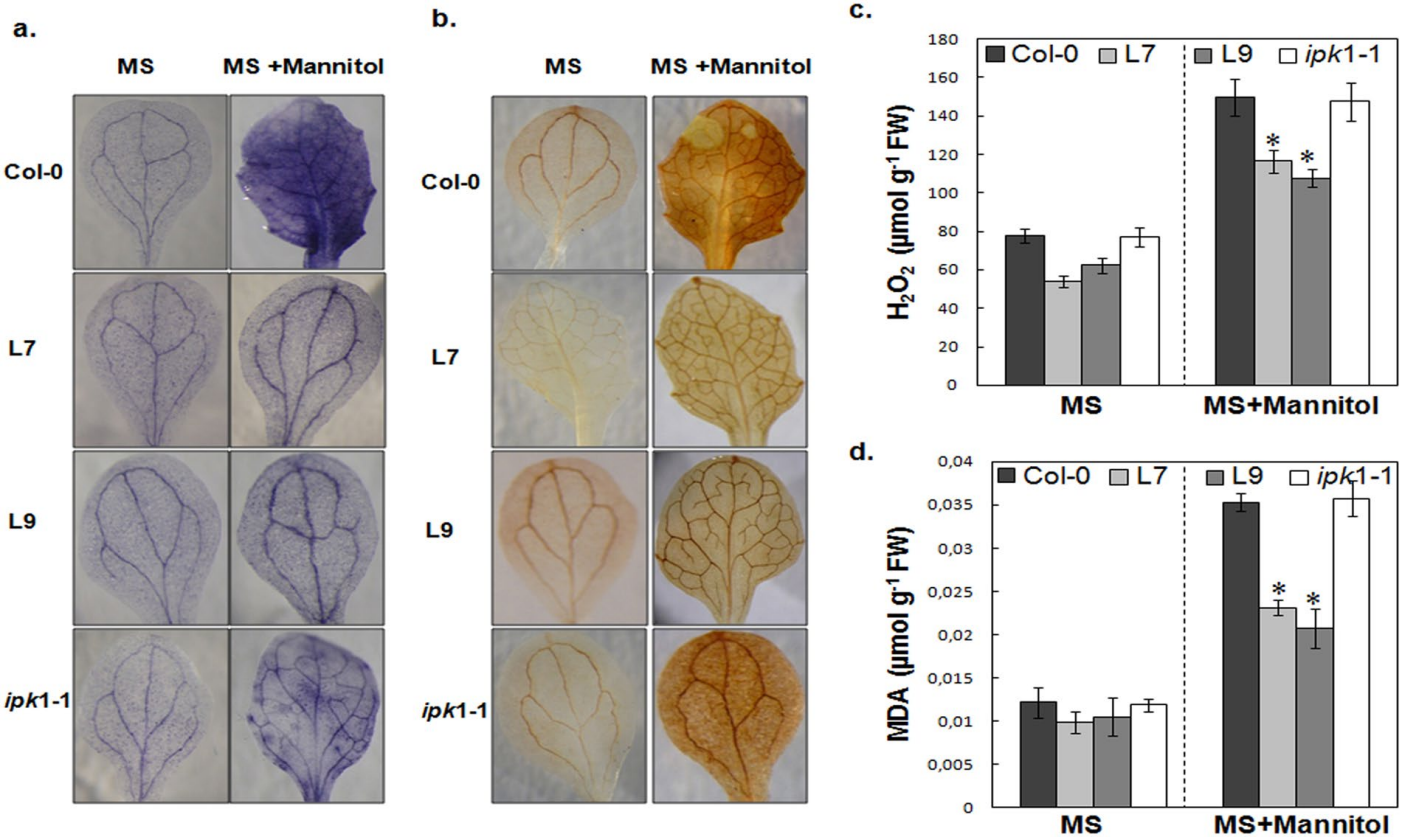
Analysis of reactive oxygen species (ROS) scavenging ability, hydrogen peroxide and malondialdehyde contents in wild-type plants (Col-0), transgenic lines (L7 and L9) and *ipk1-1* mutant. The leaves of WT, transgenic lines and *ipk*1-1 mutant were analyzed by staining with nitrobluetetrazolium (NBT) and 3,3′‐Diaminobenzidine (DAB) to reveal accumulation of O_2_^−^ radicals (**a**) and hydrogen peroxide (H_2_O_2_) (**b**). H_2_O_2_ (**c**) and malondialdehyde (MDA) (**d**) contents in WT, L7, L9 and *ipk*1-1 mutant under osmotic stress. Seeds were sown on MS medium for 7d for germination and then were transferred into MS medium or MS medium supplied with 300 mM mannitol for additional one week. Mean ± SD is shown for three independent replicates. Asterisks indicate a statistically significant difference (*P < 0.05) relative to WT (Col-0).

Previous research results indicate that various abiotic stresses induce the production of ROS leading to oxidative stress^60^ that can cause cellular damages such as lipid peroxidation^61^. Nevertheless, plants have developed an antioxidant defense system to protect cells against such oxidative damages by scavenging of ROS^58,62,63^. The observed higher foliar levels of AsA (Fig. 3b), in the L7 and L9 transgenic lines prompt us to assess whether these PHY-transgenic lines accumulate less ROS. Therefore, we performed two distinct histochemical staining assays on leaves to detect two main ROS species, O_2_^−^ and H_2_O_2_ using NBT (blue) and DAB (brown) respectively (Fig. 4). Under standard condition (MS), all tested seedlings showed a weak NBT and DAB stainings (Fig. 4a and b). Nevertheless, upon exposure to osmotic stress (300 mM mannitol), intense stainings were observed on leaves of WT and *ipk*1-1 mutant, but not in L7 and L9 lines that exhibited a weak staining comparable to the unstressed transgenic seedlings. In agreement with histochemical stainings, we noticed significantly lower levels of H_2_O_2_ in L7 and L9 transgenic lines (24%–29% less than WT) under osmotic stress conditions, compared to WT plants and *ipk*1-1 (Fig. 4c).

To further corroborate the attenuation of oxidative stress in the PHY-transgenic L7 and L9 lines, the levels of malondialdehyde (MDA), one of the products of membrane lipid peroxidation, were determined. As shown in Fig. 4d, the MDA contents of unstressed plants showed no obvious differences between the different genotypes tested (WT, L7 and L9, and *ipk1-1*). Nevertheless, when exposed to osmotic stress, the MDA contents markedly increase in WT and *ipk1-1* mutant with a 3-fold higher compared to plants grown under standard condition (MS) (Fig. 4d). In the case of L7 and L9 transgenic lines, there is an increase of the MDA contents but they remain significantly lower than in WT (Fig. 4d).

In contrast to WT and *ipk1-1* mutant, our results provide evidence for higher capacity of the L7 and L9 transgenic lines to detoxify ROS and alleviate lipid peroxidation, likely due to higher accumulation of AsA contents.

### The PHY-US417 transgenic plants exhibit an activation of enzymatic antioxidant systems

**Figure 5:**
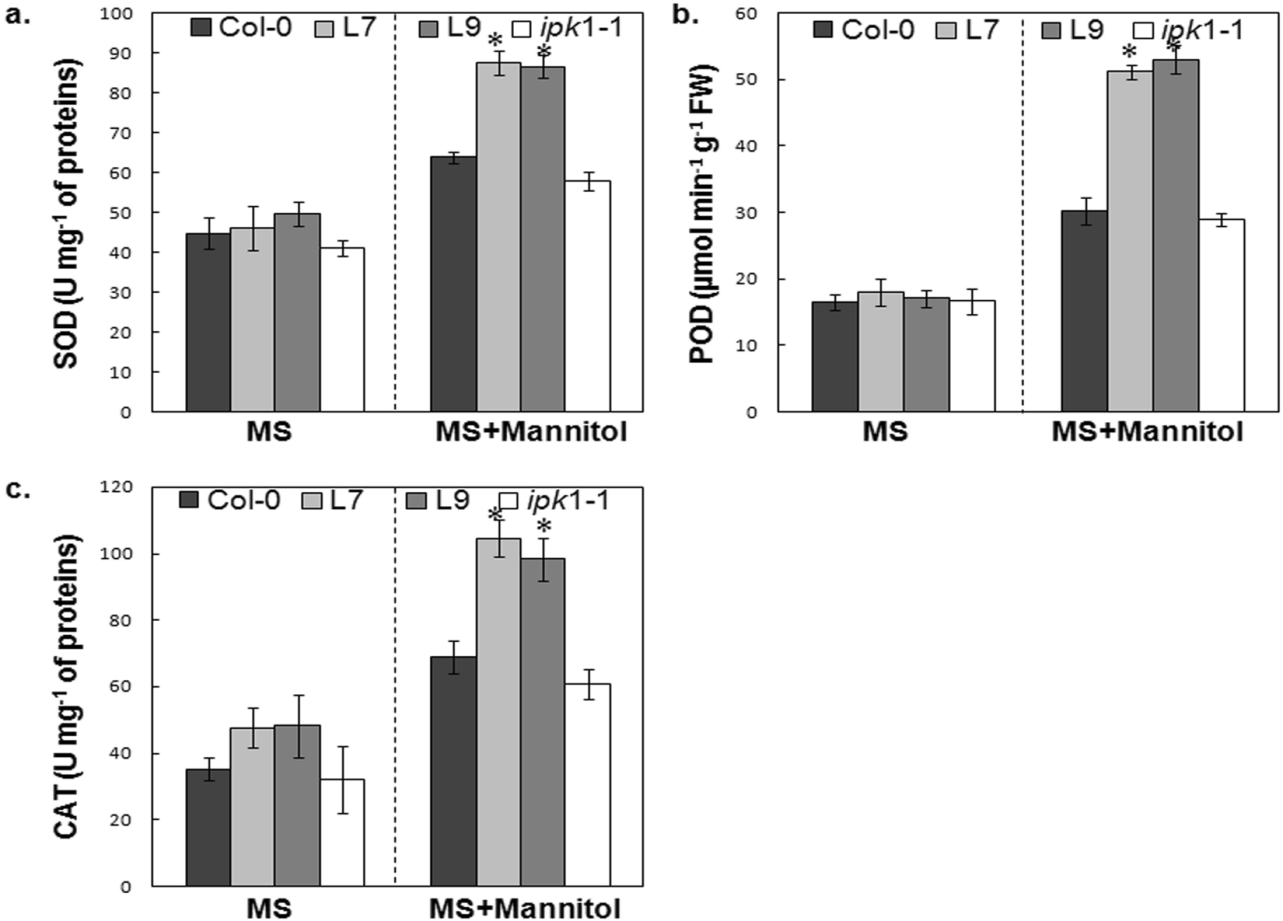
Analysis of enzymatic activities of superoxide dismutase, peroxidase and catalase in wild-type plants (Col-0), transgenic lines (L7 and L9), and *ipk*1-1 mutant under osmotic stress. Seeds were sown for 7d for germination and then were transferred into MS medium in the absence or the presence of 300 mM mannitol. After one week of treatment, seedlings were evaluated for enzymatic activities: superoxide dismutase (SOD) (**a**); Peroxidase (POD) (**b**); catalase (CAT) (**c**). Mean ± SD is shown for three independent replicates. Asterisks indicate a statistically significant difference (p < 0.05) relative to WT (Col-0).

Given the higher capacity of PHY overexpressing lines to attenuate oxidative stress, we were then interested to determine whether their osmotolerance is associated to an activation of the enzymatic antioxidant systems in addition to the increase in AsA contents. Therefore, we analyzed the enzymatic activities of superoxide dismutase (SOD), catalase (CAT) and total peroxidases (POD) in the leaves of L7 and L9 transgenic lines, *ipk*1-1, and WT. In absence of stress, SOD, CAT and POD activities were similar in all genotypes tested (Fig. 5). These activities increased under stress conditions, but the increment of these enzymatic activities was more pronounced in transgenic lines than in WT and *ipk*1-1mutant. We have registered in PHY-transgenic lines 38%, 53% and 65% increases in SOD, CAT and POD activities respectively, compared to WT. These results further support our finding that the overexpression of PHY-US417 in *Arabidopsis* plants results in a higher induction of the antioxidant systems (enzymatic and non-enzymatic antioxidants) under osmotic stress, thus contributing to restrict the ROS levels and to alleviate lipid peroxidation. Taken together, these characteristics of L7 and L9 PHY-transgenic lines lead us to suggest that decreasing PA levels can be an attractive biotechnological way to devise new strategy for enhancing plant tolerance to salt and osmotic stress.

### The phytase PHY-US417 over-expression leads to an enhanced tolerance to combined osmotic and mineral stress treatments

**Figure 6:**
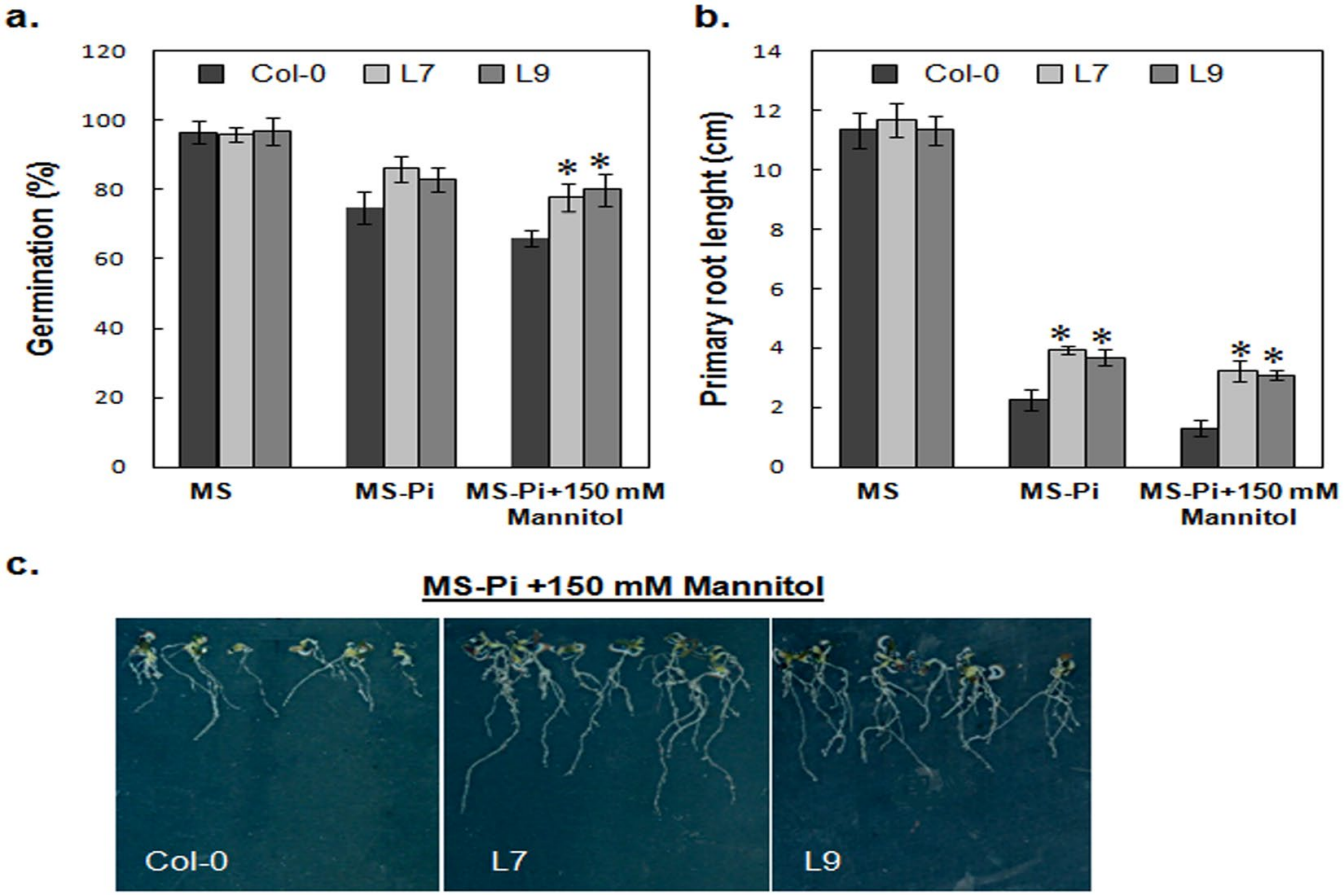
Response of wild-type plants (Col-0) and transgenic lines (L7 and L9) to phosphate deficiency or combined phosphate and mannitol stresses. (**a**) Seed germination percentage on Pi deficiency (-Pi) individual stress or Pi deficiency (-Pi)/150 mM mannitol combined stress. Vernalized seeds of WT, transgenic and mutant plants were sown in triplicates then monitored for 5 days. Seedlings with green cotyledons were counted for 100 seeds of each genotype. Data are mean ± SD of three independent experiments. (**b**) Root lengths were measured on WT (Col-0) and transgenic lines (L7 and L9) which were grown as indicated in a. (**c**) Photographs of representative WT (Col) and transgenic lines taken 2 weeks after transfer to MS medium without phosphate supplemented with 150 mM mannitol. Data are mean ± SD of three independent experiments. Asterisks indicate a statistically significant difference (p < 0.05) relative to WT (Col-0).

Finally and as plants face in their natural environments multiple stresses, we monitored the response of the PHY-overexpressors to combined stress treatments (-Pi/150 mM mannitol). Our results show that the WT control plants are severely affected under these conditions, with seed germination rates exceeding merely 60% (Fig. 6a). In addition the germinated seedlings appear small with short roots and they stop growing within two weeks. Whereas, the transgenic lines L7 and L9 although affected can clearly better withstand the adverse effects of both stresses (Fig. 6).

## Discussion

In their natural habitat, plants have to cope with multiple stresses simultaneously, such as nutrients limitation (*e.g*. Pi deficiency) and salinity in soil. It is well known that plants have evolved different mechanisms to control either nutritional^64^ or cellular osmotic status^4^. Emerging research data indicates that these mechanisms may interact, but how the different signaling pathways interact, remains poorly understood. In this study, we demonstrated that a moderate (40%) decrease of a key Pi-contain component (PA) levels by overexpressing the phytase PHY-US417 in *Arabidopsis* results into (i) an alleviated oxidative stress status, (ii) lower sensitivity to ABA and (iii) an enhanced osmotic stress tolerance. In combination with our previous data in which we showed that lowering the in L7 and L9 plants improves their capacity to respond to Pi deficiency^51^, here we demonstrated that lowering PA confers plants tolerance to additional environmental stresses, i.e. osmotic stress. Interestingly, even under severe stress conditions by exposing plants to combined stress treatments (-Pi and mannitol), the PHY-transgenic lines show clearly a better survival rates compared to WT plants. These multiple fitness benefits obtained by overexpressing the phytase PHY-US417 in plants constitute a desired characteristic for transgenic crop plants nowadays.

PA was proposed to be involved in various signaling pathways in plants, ranging from nutritional (Pi)^65^, to hormonal (auxin and jasmonate)^66,67^ responses. Thus it is not surprising to see that a strong decrease of PA concentration in plants, through the mutation of genes involved in its biosynthesis pathways (e.g IPK1), causes a severe growth defect^13,41,44,48^. But, interestingly, a moderate decrease of PA, such as the level obtained through the expression of bacterial phytases, shows rather a positive effect on plant growth under abiotic stress. Indeed, in contrast to *ipk1-1*, the overexpression of PHY-US417 in L7 and L9 lines was shown to cause a decrease of PA that not only does not alter plant germination and plant growth^51^, but also increases their capacity to respond to Pi deficiency stress by showing a better growth capacity. Remarkably, while Pi deficiency is known to inhibit the primary root growth in wild-type plants, L7 and L9 lines exhibited longer roots under this stress (-Pi) condition^51^. In this study, our results showed that decreasing PA in L7 and L9 transgenic lines confers plants with a new characteristic, a higher tolerance to osmotic stress and lower ABA sensitivity. This was revealed by higher germination rates and enhanced root growth of L7 and L9 under salt and osmotic stresses, compared to *ipk1-1* mutant (Figs 1 and 2). These results are in favor of a model according to which a moderate decrease of the PA concentration confers a plant salt/osmotic stress tolerance. In literature there is growing body of evidences indicating the presence of an interesting link between Pi homeostasis and salt stress. First, *Arabidopsis* mutant in *PHO2* which leads to overaccumulate Pi in the shoots tolerate salt stress^14^. Second, some *Arabidopsis* ecotypes that show a higher salt stress tolerance are known to tolerate Pi deficiency^68,69^. More interestingly, some *Arabidopsis* accessions such as Landsberg *erecta* (Ler) that have been shown to accumulate low PA^70^ exhibited a better salt stress^68^ showing large rosette sizes in control and salt stress conditions^68^. Ler is known to tolerate Pi deficiency by developing longer roots under this stress (-Pi)^69^. In contrast, accession such as Cape Verde Islands (Cvi), which is highly sensitive to Pi deficiency and showed short root under -Pi^69^ has higher PA content than Ler-0^70^ and showed intermediate rosette weight and area under stress conditions^68^. This data suggest a genetic basis for the interaction between PA homeostasis and salt/osmotic stress tolerance. In future research work, comparative genome wide association mapping (GWAS) will give the opportunity to identify gene(s) that associate plant growth under salt/osmotic stress and PA content. Worth to note that the degradation of PA by PHY-US417, which as a β−propeller phytase, is expected to sequentially remove only 3 Pi groups would result in the release of myo-inositol triphosphates^71,72^. Therefore, we cannot rule out possible involvement of such inositol phosphate intermediates in stress signaling pathways including osmotic stress response. However, the identity of these Pi-compounds and how they would influence signaling pathways remains an open question.

Moreover, and in contrast to *ipk1-1*, L7 and L9 transgenic lines showed lower ABA sensitivity. Decreases in ABA sensitivity in the PHY-overexpressors may be linked to the intracellular levels of *myo*-inositol and a relationship between ABA and *myo*-inositol, as such was already evoked. In *Arabidopsis* the mutation of the *MIPS1* gene (encoding the L-*myo*-inositol 1-phosphate synthase that catalyzes the rate-limiting step in the synthesis of myo-inositol) results in lower *myo*-inositol levels and increased ABA sensitivity^73^. Similarly, rice transgenic lines where the MIPS gene was down regulated by seed-specific RNAi silencing, exhibit lower *myo*-inositol contents and altered ABA sensitivity^31^. Therefore these observations raise a possible involvement of *myo*-inositol in regulating plant ABA response. Nonetheless, *myo*-inositol might be not the most critical component and other related molecules including phytate itself might be even more decisive in ABA signaling. Such an assumption is reinforced by the finding of Lemtiri-Chlieh showing that IP_6_ mimics the inhibitory effect of ABA on the K^+^-inward current in guard cells of *Solanum tuberosum* and *Vicia faba*^74^.

In plants, tolerance to osmotic stress can be associated to a higher level of AsA and to a lower membrane damage and ROS accumulation^58,63^. As an antioxidant, AsA is known to play a significant role in plant survival under stress conditions and has the ability to restrict the levels of ROS and lipid peroxidation^75,76^. These results are in agreement with our results showing that the osmotolerance observed in L7 and L9 is associated to higher accumulation of AsA (Fig. 3b). Similar results were also reported by Zhang *et al*.^77^, who showed that overexpression of the purple acid phosphatase AtPAP15 phytase leads to increased tolerance of *Arabidopsis* to salt and osmotic stresses and that this improvement is also correlated with decreased foliar phytate and increased foliar AsA levels. A working model was proposed in which the degradation of PA by AtPAP15 allows the production of *myo*-inositol which then can serve as an entry point into AsA biosynthesis^53,77^. It is noteworthy that not only AsA levels increase but also enzymatic antioxidant (CAT, SOD and POD) activities are enhanced in transgenic lines. Higher level of antioxidative enzymes was detected under osmotic stress. This could decrease the accumulation of ROS and be responsible for enhanced tolerance abiotic stresses in transgenic *Arabidopsis*. How the overexpression of PHY-US417 leads to an activation of enzymatic antioxidant systems remains an intriguing question. Thus plant osmotic stress tolerance in the transgenic lines expressing the phytase can occur presumably via stimulation of enzymatic and non enzymatic antioxidant systems.

In conclusion, our previous results^51^ together with the current study show that overexpression of a bacterial phytase PHY-US417 offers *Arabidopsis* plants several beneficial traits: enhanced tolerance to Pi deficiency^51^ and osmotic stress, either individual or combined stresses. Such a characteristics offred by a single transgene provides plants with clear advantage to better grow in their natural environment often having low P but high salt concentrations.

## Methods

### Plant material and growth conditions

Fresh seeds of WT *Arabidopsis thaliana* ecotype Columbia (Col-0), transgenic lines expressing *PHY-*US417^51^ and the *ipk*1-1 mutant^35^ were collected from plants grown side by side under the same standard growth conditions. Seeds were surface sterilized with 70% ethanol for 10 min, rinsed with water three times and plated onto Murashige and Skoog (MS) media^78^ without or with 125 mM NaCl (Sigma), 150 mM mannitol (Sigma) or increasing concentrations (1, 2.5 and 5 µM) of abscisic acid ABA (Sigma). Seedlings were grown for two weeks on vertical plates under light/dark cycle conditions of 16/8 h, light intensity of 250 µmol m^−2^ s^−1^, and temperature of 22–24 °C in growth chamber. For growth assessment under osmotic stress treatments, one-week-old seedlings were transferred onto MS agar media only or containing 300 mM mannitol for 2 additional weeks. Plates were grown as aforementioned. After photo documentation, root lengths were measured using the OPTIMAS software (OPTIMAS Image Analysis system 6.1, Media Cybernetic). Then, shoots were collected for measurements of AsA, chlorophyll, H_2_O_2_ and MDA levels as well as antioxidant enzymatic activities (see below).

### Ascorbate content determination

Plant tissues were homogenized in ice-cold 6% trichloroacetic acid (TCA) (Sigma), the homogenate centrifuged at 6000 rpm for 25 min at 4 °C, and the supernatant was collected. AsA contents were measured by a modified version of the procedure described by Gillespie and Ainsworth^79^ based on an original method developed by Masato *et al*.^80^. In this assay, ferric ion (Fe^3+^) is reduced by AsA to the ferrous ion (Fe^2+^), which when coupled with 2,2′-dipyridyl forms a complex with characteristic absorbance at 525 nm. The AsA concentration was expressed in nmol AsA per well according to the standard curve generated using known concentrations of AsA. The value was then converted to µmol g^−1^ tissue fresh weight.

### Measurement of chlorophyll contents

After osmotic stress treatment (300 mM mannitol), fresh leaves (100 mg) of *Arabidopsis* plants were collected to determine the chlorophyll contents. Chlorophyll was extracted using 80% acetone and then the homogenates were centrifuged at 12,000 rpm for 10 min at 4 °C. The absorbance of the obtained supernatants was measured in a spectrophotometer at 646 nm and 663 nm and total chlorophyll content in each sample, was calculated according the following formula: [(7.15 × DO663) + (18.71 × DO646)]V/M, where V corresponds to the volume of added acetone and M to the weight of fresh material (FW).

### Determination of proline content

Proline content was measured by the method of Bates *et al*.^81^. Plant tissue (0.1 g) was homogenized in 40% methanol solution. The homogenate was heated in a water bath at 85 °C for 1 h. Tubes were covered with aluminum foil to avoid polarization of alcohol and then cooled. After addition of acid-ninhydrin solution, mixtures were boiled during 30 min and placed on ice to stop the reaction. The chromophore obtained was extracted from liquid phase with toluene (v/v) and the absorbance of organic layer was read at 520 nm (JENWAY 7305 Spectrophotometer). Proline concentration was determined from calibration curve using L-Proline as standard (proline solutions ranging from 0.04 to 1 mM) and expressed as µmol proline g^−1^ FW.

### Detection of ROS (O_2_^−^ and H_2_O_2_) species

*Arabidopsis* plants treated or not with 300 mM mannitol were harvested for staining with nitrobluetetrazolium (NBT) or 3,3′‐Diaminobenzidine (DAB). For detection of superoxide anion (O_2_^−^), seedlings were transferred in 1 mg.ml^−1^ fresh NBT solution (prepared in 25 mM HEPES, pH 7.6) and infiltrated in a vacuum for 5 min. Then, the plants were incubated under dark conditions for 2 h followed by a treatment with 80% ethanol. For hydrogen peroxide (H_2_O_2_) detection, seedlings were treated with 1 mg/ml DAB solution (prepared in 10 mM Na_2_HPO_4_ solution, pH 3.8) and subjected to vacuum infiltration for 5 min. After a dark incubation for 4 h under stirring, plants were bleached in ethanol: acetic acid: glycerol solution (3:1:1). In both cases, stained leaves were photographed with a Leica MS5 stereomicroscope.

### Measurement of hydrogen peroxide content

The levels of hydrogen peroxide (H_2_O_2_) were measured according to the method of Alexieva *et al*.^82^. Fresh shoot tissues (0.2 g) were homogenized with 0.1% (w/v) trichloroacetic acid (TCA) and were centrifuged at 12,000 *g* for 15 min at 4 °C. Supernatant (0.5 ml) was added to 0.5 ml of 10 mM potassium phosphate buffer (pH 7.0) and 1 ml of 1 M potassium iodide (KI). Finally, the absorbance of the reaction mixture was measured at 390 nm. The amount of H_2_O_2_ was calculated using a standard curve prepared from known concentrations of H_2_O_2_ ranging from 0.1 to 1 mM.

### Determination of lipid peroxidation

Lipid peroxidation was estimated by measuring malondialdehyde (MDA) content in *Arabidopsis* seedlings subjected or not to osmotic stress (300 mM mannitol) following the procedure described by Heath and Packer^83^. 0.1 g of seedlings were homogenized in 0.1% TCA and mixed with 0.5% thiobarbituric acid (TBA). The homogenate was heated at 95 °C for 30 min, then cooled on ice to stop the reaction and centrifuged at 10,000 *g* for 10 min. Absorbance of the supernatant was measured at 532 nm and corrected for non-specific turbidity by subtracting the absorbance at 600 nm. The concentration of MDA was calculated by using an extinction coefficient of 155 mM^−1^ cm^−1^.

### Protein extraction and antioxidant enzyme activities

Plant tissue (0.5 g) was ground in liquid nitrogen and homogenized in cold solution containing 100 mM Tris-HCl buffer (pH 8.0), 10 mM EDTA, 50 mMKCl, 20 mM MgCl_2_, 0.5 mM Phenylmethylsulfonyl fluoride (PMSF), and 2% (w/v) Polyvinylpyrrolidone (PVP). The homogenate was centrifuged for 20 min at 10,000 g and at 4 °C and then the supernatant was collected for the determination of antioxidant activities. Protein concentration was determined according to Bradford^84^ using bovine serum albumin as a standard.

Superoxide dismutase (SOD) activity was determined by monitoring the inhibition of photochemical reduction of nitrobluetetrazolium (NBT) according to the method employed by Rao *et al*.^85^. An aliquot of enzyme extract was mixed with reaction buffer solution (2.6 mM Na_2_EDTA**-**methionine (pH 7.0), 4.4 mM nitroblue tetrazolium and 2 μM riboflavin). The reaction solution was illuminated for 20 min. A non-irradiated reaction mixture that did not develop color was used as a control. One unit of SOD activity was defined as the amount of enzyme required to cause a 50% inhibition of the reduction of NBT as monitored at 560 nm. Catalase (CAT) activity was determined spectrophotometrically by monitoring the rate of breakdown of H_2_O_2_ at OD240 as described previously^86^. An aliquot of crude enzyme extract was added to the reaction mixture containing 50 mM phosphate buffer (pH 7.0), 30 mM H_2_O_2_. Reduction in optical density of the reaction mixture at 240 nm were recorded every 20 s. Peroxidase (POD) activity was estimated by the guaiacol oxidation method according to Nickel and Cunningham^87^. The reaction mixture contained an aliquot of crude enzyme extract with 50 mM phosphate buffer (pH 7), 20 mM guaiacol and 40 mM H_2_O_2_. The decrease of the absorbance at 470 nm was recorded for 2 min. Enzyme activity, was expressed in μmol.min^−1^.g^−1^ FW and was calculated using an extinction coefficient of 26.6 mM^−1^ cm^−1^.

### Statistical analyses

Statistical analyses of the data were performed using analysis of variance (ANOVA) and the Tukey’s test to compare mean values. Only differences with p values <0.05 were considered as significant.
